# Effect of miR-134 against myocardial hypoxia/reoxygenation injury by directly targeting NOS3 and regulating PI3K/Akt pathway^1^

**DOI:** 10.1590/s0102-865020190080000002

**Published:** 2019-10-14

**Authors:** Jian-Min Xiao, Ji-Jia Wang, Li-Li Sun

**Affiliations:** I Master, Department of Cardiovascular Medicine , Daqing Oilfield General Hospital , Daqing , Heilongjiang , P.R. China . Technical procedures, interpretation of data, statistical analysis, manuscript preparation.; II Master, Department of Geriatric Medicine , Daqing Oilfield General Hospital , Daqing , Heilongjiang , P.R. China . Conception and design of the study, critical revision.

**Keywords:** Myocardial Reperfusion Injury, MicroRNAs, Nitric Oxide Synthase Type III, Apoptosis, Cell Proliferation

## Abstract

**Purpose:**

To reveal the function of miR-134 in myocardial ischemia.

**Methods:**

Real-time PCR and western blotting were performed to measure the expression of miR-134, nitric oxide synthase 3 (NOS3) and apoptotic-associated proteins. Lactic dehydrogenase (LDH) assay, cell counting kit-8 (CCK-8), Hoechst 33342/PI double staining and flow cytometry assay were implemented in H9c2 cells, respectively. MiR-134 mimic/inhibitor was used to regulate miR-134 expression. Bioinformatic analysis and luciferase reporter assay were utilized to identify the interrelation between miR-134 and NOS3. Rescue experiments exhibited the role of NOS3. The involvement of PI3K/AKT was assessed by western blot analysis.

**Results:**

MiR-134 was high regulated in the myocardial ischemia model, and miR-134 mimic/inhibitor transfection accelerated/impaired the speed of cell apoptosis and attenuated/exerted the cell proliferative prosperity induced by H/R regulating active status of PI3K/AKT signaling. LDH activity was also changed due to the different treatments. Moreover, miR-134 could target NOS3 directly and simultaneously attenuated the expression of NOS3. Co-transfection miR-134 inhibitor and pcDNA3.1-NOS3 highlighted the inhibitory effects of miR-134 on myocardial H/R injury.

**Conclusion:**

This present work puts insights into the crucial effects of the miR-134/NOS3 axis in myocardial H/R injury, delivering a potential therapeutic technology in future.

## Introduction

Myocardial infarction (MI) is one of the leading causes for high mortality worldwide and usually poses a huge threat to human health ^1^ . It refers to a kind of acute coronary syndrome induced by abnormal blood flow in the heart ^2 , 3^ . Prior studies have validated the importance of reperfusion as an efficacious strategy against MI ^4^ . However, the conduction of reperfusion often results in a myocardial ischemia-reperfusion (I/R) injury by diverse physiological processes ^5^ . Thus, how to prevent cardiomyocytes from I/R injury is the key for treatment of myocardial ischemia and other related diseases.

Up to date, researches have clarified that microRNAs (miRNAs) are involved in the processes of diseases caused by ischemia, regulating the posttranscription of their target genes ^6^ . A strong relationship between the level of miRNAs and disorders has been reported in the literature. For example, miR-145-5p triggers apoptosis by inhibiting dual specificity phosphatase 6 (DUSP6) after I/R ^7^ . MiR-370 has a protective effect on myocardial H/R injury in mice by modulating PLIN5-dependent PPAR signaling pathway ^8^ . Zhu *et al* . ^9^ expounded that inhibition of miR-320 enforces protective influence on myocardial I/R injury through stimulating nuclear factor NF-E2 related factor 2 (Nrf2) expression. These reports establish a foundation, implying that miRNAs can be regarded as biomarkers to apply in the treatment of myocardial I/R injury. In these miRNAs, it has been proved that miR-134 participates in neuronal cell death caused by I/R injury ^10^ . Zhou *et al* . ^11^ evaluated that the occurrence of acute ischemic stroke is associated with increased miR-134. The proliferative ability of cardiomyocyte progenitor cells could be modulated by miR-134 ^12^ . Additionally, miR-134-5p has been regarded as a promising biomarker for acute myocardial infarction diagnosis ^13^ . These researches suggested that miR-134 might hold a potent regulation of myocardial H/R injury. However, the potential effects about miR-134 have not elaborated clearly.

This present study was conducted to detect the role of miR-134 through establishing a model of myocardial H/R injury in rat cells. We assessed the expression of miR-134, finding that miR-134 has an important role on myocardial H/R injury-induced cell apoptosis and proliferation as well as lactic dehydrogenase (LDH) activity. Western blot examination revealed that inhibition of miR-134 can be used to activate the PI3K/Akt pathway. Moreover, prediction of bioinformatics software and luciferase reporter assay identified that nitric oxide synthase 3 (NOS3) is a target gene of miR-134; thereby, further experiments showed that miR-134 can attenuate the expression of NOS3 and overexpressed NOS3 was capable to intensify the influence relying on miR-134 inhibitor in myocardial I/R injury. These results introduce to us novel clues against myocardial I/R injury.

## Methods

### Myocardial ischemia model in rat cardiomyoblasts H9c2 cells

The rat cardiomyoblasts H9c2 cells were obtained from the American Type Culture Collection (Manassas, VA, USA) and then incubated in Dulbecco’s modified eagle’s medium (DMEM; Carlsbad, CA, USA) supplemented with 10% fetal bovine serum (FBS; Sigma-Aldrich, MO, USA) and gentamicin (Sigma-Aldrich). After the conversion of DMEM medium into serum-free medium, cells were exposed to anoxic environment (95% N _2_ , 5% CO _2_ and 0% O _2_ ) at 37°C for 6h. Then, reperfusion progression was performed in normal incubator with 95% O _2_ and 5% CO _2_ for additional 24 h at 37°C.

### Transient transfection

GenePharma (Shanghai, China) delivered miR-134 mimic (5’-UGUGACUGGUUGACCAGAGGGG-3’)/inhibitor (5’-CCCCUCUGGUCAACCAGUCACA-3’) and their corresponding negative control (NC; mimic NC, 5’-UUGUACUACACAAAAGUACUG-3’; inhibitor NC, 5’-CAGUACUUUUGUGUAGUACAA-3’) and the Lipofectamine 3000 (Thermo Fisher Scientific, Waltham, MA, USA) was used to implement transient transfection as the manufacturer’s instruction. The vector of pcDNA3.1-NOS3 was constructed to overexpress NOS3. Afterwards, 48h-transfected cells were applied in future experiments.

### Cell apoptosis assay

Cell apoptotic activity was assessed utilizing Annexin V/FITC kit (Beyotime, Shanghai, China). Briefly, harvested cells were put in centrifuge tubes to centrifuge two times at 1000 rpm for 5 min. Removed supernatant and resuspended by 1 × binding buffer to adjust cell density into 1-5 × 10 ^6^ /mL. Subsequently, 100 μL of cell suspension and 5 μL of Annexin V/FITC were well mixed to block in darkroom for 5 min. For machine testing, cells were susceptible to the staining mixture, 10 μL of PI and 400 μL of PBS. Finally, the results were analyzed by Flowjo software (Tree Star Inc, Ashland, OR).

### Hoechst 33342/PI double staining assay

Hoechst 33342/PI double staining was conducted according to the manufacturer’s protocols. H9c2 cells were incubated with Hoechst 33342 (10 μg/mL) and PI (10 μg/mL) at 37°C for 15 min, respectively. After washing using PBS, the cells were observed under the fluorescent microscope.

### CCK-8 assay

Cell proliferative ability was elevated by CCK-8 assay. Following transfection, cells were placed in 96-well plates with the density of 1000 cells per hole. The culture condition is 5% CO _2_ and 37°C. Next, we selected cells that cultured for 0, 24, 48 and 72h to measure corresponding proliferative prosperity. Before detection, 10 μL of CCK-8 regent were added into per well and then cells were cultured for 1.5h at 37°C. The wavelength of 450 nm was set up for optical density (OD) value examination using a microplate reader.

### RNA isolation and quantitative real-time PCR

To isolate total RNA, TRIzol (Invitrogen, Carlsbad, CA, USA) was presented according to the manufacturer’s protocols. RNA of specific gene was reverse transcribed into complementary DNA (cDNA) by means of PrimeScript RT Reagent Kit (Takara, Japan). Real-time PCR was conducted at 7900HT real-time PCR system using SYBR Premix Ex Taq II (Takara, Japan). By contrast, the conversion of miRNA into cDNA was assisted by the MiScript Reverse Transcription kit (Qiagen GmbH, Hilden, Germany). Correspondingly, the expression level of miR-134 was confirmed via MiScript SYBR-Green PCR kit (Qiagen). GAPDH and U6 were normalized to experimental control and the 2 ^-^ method was applied to calculate relative expression level of specific gene or miRNA.

The primer sequences are as follows:

**Table:** 

NOS3	forward primer, 5’-GTGATGGCGAAGCGAGTGAAG-3’;
	reverse primer, 5’-CCGAGCCCGAACACACAGAAC-3’.
GAPDH	forward primer, 5’-GTCTCCTCTGACTTCAACAGCG-3’;
	reverse primer, 5’-CCGAGCCCGAACACACAGAAC-3’.
miR-134	sense: 5’-TGTGACTGGTTGACCAGAGG-3’;
	antisense: 5’-GAACATGTCTGCGTATCTC-3’.
U6	sense: 5’-CTCGCTTCGGCAGCACA-3’;
	antisense: 5’-AACGCTTCACGAATTTGCGT-3’.

### Protein extraction and western blot

Protein was extracted relying on RIPA lysate with protease inhibitor and their concentration was revealed by the bicinchoninic acid (BAC) method. After being denatured at 95°C for 5 min, protein samples were separated in 12% SDS-PAGE and transferred to PVDF membranes. This was followed by incubating in 5% skim milk for 1h and blocking with primary antibodies (1:1,000; Cell Signaling Technology, Danvers, MA, USA) overnight. Subsequently, the membranes were washed using TBST and incubated in secondary antibodies. The addition of ECL contributed to develop and QUANTITY ONE software was to scan gray values of protein bands. The primary antibodies contain Bcl-2, Cleaved Caspase-3, Cleaved Caspase-9, NOS3, PI3K, p-PI3K, Akt, p-Akt and GAPDH.

### Lactic dehydrogenase (LDH) detection

After the H/R treatment, the culture medium was removed and pre-cold PBS was utilized to wash cells. Extract regent was added according to the number of cells (10 ^4^ ) and the volume of extract (mL) at a ratio of 500:1. Then, cells were disrupted on ice, centrifuged at 8000g for 10 min at 4°C. LDH levels of cell supernatant were immediately detected with LDH kit (Solarbio, Beijing, China) under a spectrophotometer.

### Luciferase reporter assay

The HEK 293T cells were seeded into 24-well plates. When cell confluence was up to 80%, NOS3-wild type (NOS3-WT) and NOS3-mutant (NOS3-MUT) with miR-134 mimic were co-transfected. At 48 h post transfection, proteins were isolated and luciferase activity was determined with Dual-Luciferase Reporter Assay Kit (Promega, Madison, WI).

### Data analysis

All experiments were repeated at least three times and data were presented as means ± standard deviation (SD). The comparison in two groups was analyzed using t-test while multiple comparisons were demonstrated by one-way analysis of variance (ANOVA) along with post hoc test, Tukey or Dunnett. The statistical software included SPSS22.0 and GraphPad Prism 6.0. P < 0.05 was regarded as significant criteria.

## Results

### MiR-134 was upregulated caused by hypoxia/reoxygenation (H/R) and transient transfection efficiency

**Figure 1:**
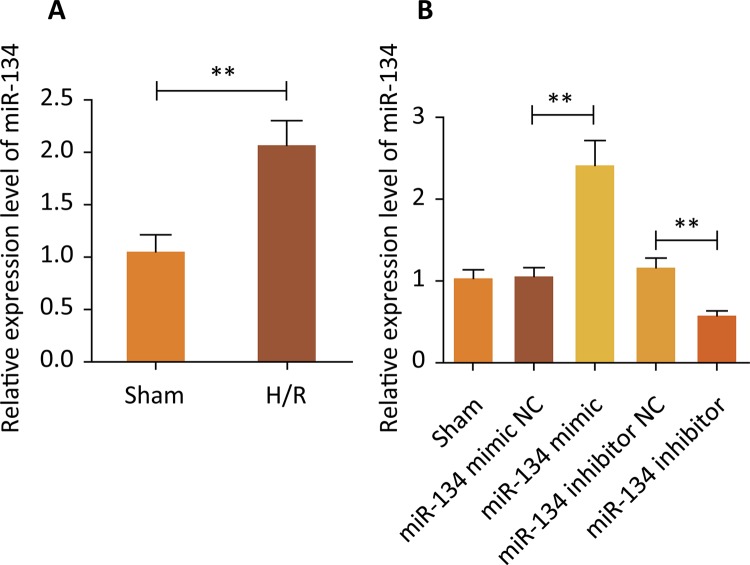
- Expression of miR-134 in H9c2 cells under H/R environment. ( A ) The expression of miR-134 was higher in the H/R group than that of the Sham group, **P<0.01 *vs.* Sham group. ( B ) After different treatments, the miR-134 expression level in H9c2 cells, **P<0.01 *vs.* Sham group.

To explore the function of miR-134 in myocardial ischemia, an in vitro myocardial ischemia model was constructed by treating rat cardiomyoblasts H9c2 cells with H/R conditions. The mRNA analysis of treated H9c2 cells, showed significant increases of miR-134 expression level in the H/R group compared with the Sham group ( Fig. 1A , P<0.01). Next, in preparation for the later trial, miR-134 mimic and miR-134 inhibitor were used to transfect H9c2 cells, respectively. As can be seen from Figure 1B , compared with miR-134 mimic/inhibitor NC, miR-134 showed a higher level because of the interference of miR-134 mimic while miR-134 revealed a lower level caused by miR-134 inhibitor (P<0.01).

### Deletion of miR-134 mitigates myocardial injury induced by H/R

**Figure 2:**
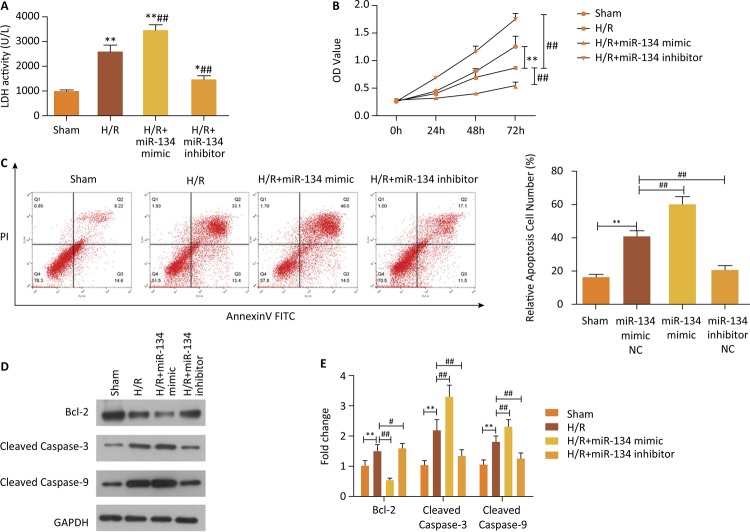
Cell apoptosis caused by H/R was enhanced with miR-134 mimic transfection but blocked via miR-134 inhibitor intervention. ( A ) LDH activity was examined in all groups, **P<0.01 *vs.* Sham group, ## P<0.01 *vs.* H/R group. ( B ) Cell proliferation was explored by CCK-8, **P<0.01 *vs.* Sham group, ## P<0.01 *vs.* H/R group. ( C ) The effects of mR-134 on cell apoptotic ability by the means of flow cytometry assay and quantified, **P<0.01 *vs.* Sham group, ## P<0.01 *vs.* H/R group. ( D ) and ( E ) The apoptosis-related proteins were assessed with western blotting, **P<0.01 *vs.* Sham group, # P<0.05 *vs.* H/R group, ## P<0.01 *vs.* H/R group.

LDH detection, cell proliferation assay and flow cytometry examination were performed to identify the effect of miR-134 on myocardial injury. LDH activity was higher in cells from the H/R group than from the Sham group, which indicated consistent pattern in H/R+miR-134 mimic group, while the H/R+miR-134 inhibitor group demonstrated the lower levels of LDH ( Fig. 2A , P<0.05). Moreover, miR-134 mimic transfection exacerbated myocardial injury via encouraging cell apoptosis and impairing cell viability ( Fig. 2B-C , P<0.01). Another important finding was that the transfection of the miR-134 inhibitor had a diametrically opposite effect on cell apoptosis and growth compared to miR-134 mimic infection ( Fig. 2B-C , P<0.01). The following findings were consistent with the above mentioned observation that significant the reduction of the Bcl-2 protein expression was observed in the H/R+miR-134 mimic group when compared with that in the H/R and Sham groups ( Fig. 2D-E , P<0.05). Meanwhile, pro-apoptotic proteins, Cleaved Caspase-3 and Cleaved Caspase-9, were evidently increased ( Fig. 2D-E , P<0.05). It can therefore be assumed that the miR-134 downregulation can lighten cardiomyoblasts injury derived from H/R.

### Inhibition of PI3K/Akt signaling pathway led by H/R was restored due to the downregulation of miR-134

**Figure 3:**
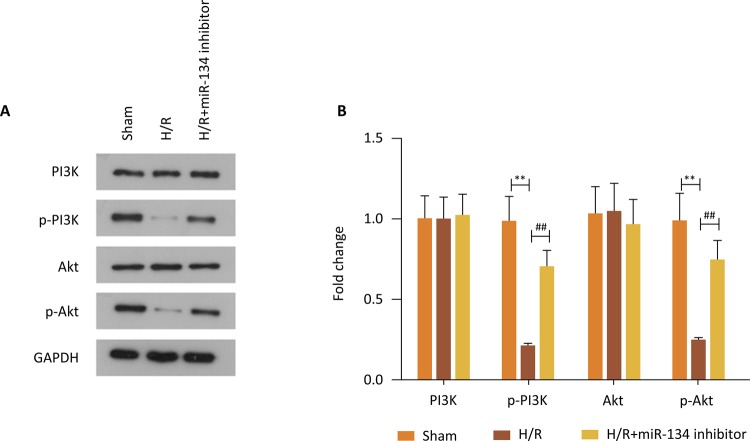
Inhibition of the PI3K/Akt signaling pathway led by H/R was restored due to the downregulation of miR-134. ( A ) and ( B ) Western blot assay was conducted to test the expression of key proteins in the PI3K/Akt signaling pathway, **P<0.01 *vs.* Sham group, ## P<0.01 *vs.* H/R group.

It is apparent true from explorations that the PI3K/Akt signaling pathway plays a protective role on cardiomyoblasts via mediating the evolution of myocardial injury. In Figure 3A , there is a clear trend showing a decreasing expression level of p-PI3K and p-Akt in H9c2 cells treated with H/R compared with control group, while PI3K and Akt showed no obvious difference. On the contrary, miR-134 knockdown restored the expression of p-PI3Kand p-Akt to some extent, manifesting the activation of the PI3K/Akt signaling pathway ( Fig. 3A ). Besides, the gray values quantitation of proteins clearly revealed this influence ( Fig. 3B , P<0.01). In summary, for the information in these explanations, reduction of miR-134 can protect cardiomyoblasts from H/R-induced myocardial injury through the activation of the PI3K/Akt signaling pathway.

### NOS3 was directly targeted by miR-134 and its overexpression stresses the mitigation of miR-134 on myocardial damage

**Figure 4:**
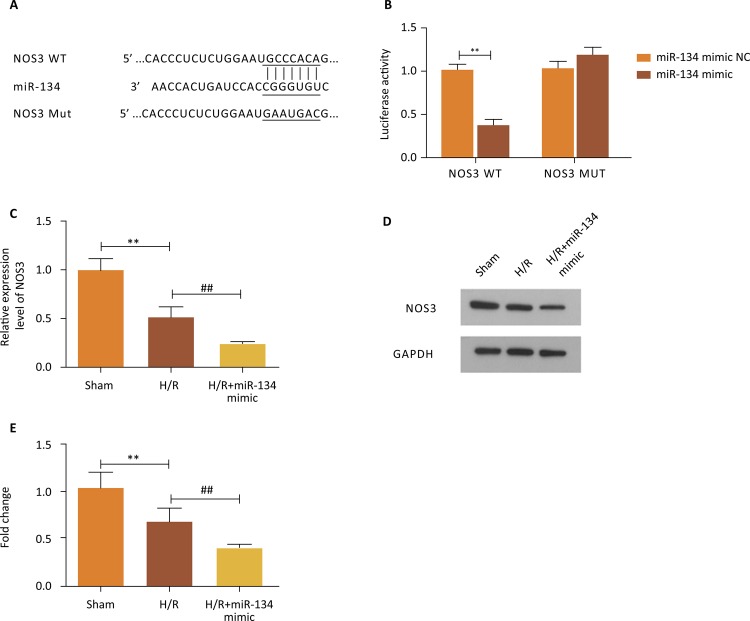
- NOS3 was directly targeted by miR-134 and simultaneously its expression was suppressed owing to the overexpression of miR-134. ( A ) The sequence of binding site between miR-134 and NOS3. ( B ) The luciferase activity was decreased because of miR-134 mimic in the NOS3-WT group but increased in the NOS3-MUT group, **P<0.01 *vs.* miR-134 mimic NC group. ( C ) The mRNA expression of NOS3 was investigated through qRT-PCR, **P<0.01 *vs.* Sham group, ## P<0.01 *vs.* H/R group. ( D ) and ( E ) Evaluation of NOS3 expression in western blot, **P<0.01 *vs.* Sham group, ## P<0.01 *vs.* H/R group.

The association between miR-134 and NOS3 was figured out subsequently. Bioinformatic software provided the complementary sequence of miR-134 and NOS3, which was exhibited in Figure 4A . By analyzing the result of luciferase reporter assay, we verified their interrelation: cells treated with NOS3-WT and miR-134 mimic delivered markedly lower luciferase activity while cells that co-transfected with NOS3-WT and miR-134 mimic presented no differences in luciferase activity ( Fig. 4B , P<0.01). Moreover, we also performed qRT-PCR and western blotting experiments to further identify this interaction. After treating with H/R environment, the relative mRNA expression of NOS3 was significantly decreased compared to the Sham group and the addition of miR-134 mimic reinforced the inhibitory effect caused by H/R ( Fig. 4C , P<0.01). In the same way, the findings of western blot and quantified analysis showed a downtrend at the NOS3 protein expression level in corresponding teams ( Fig. 4D-E , P<0.01).

**Figure 5:**
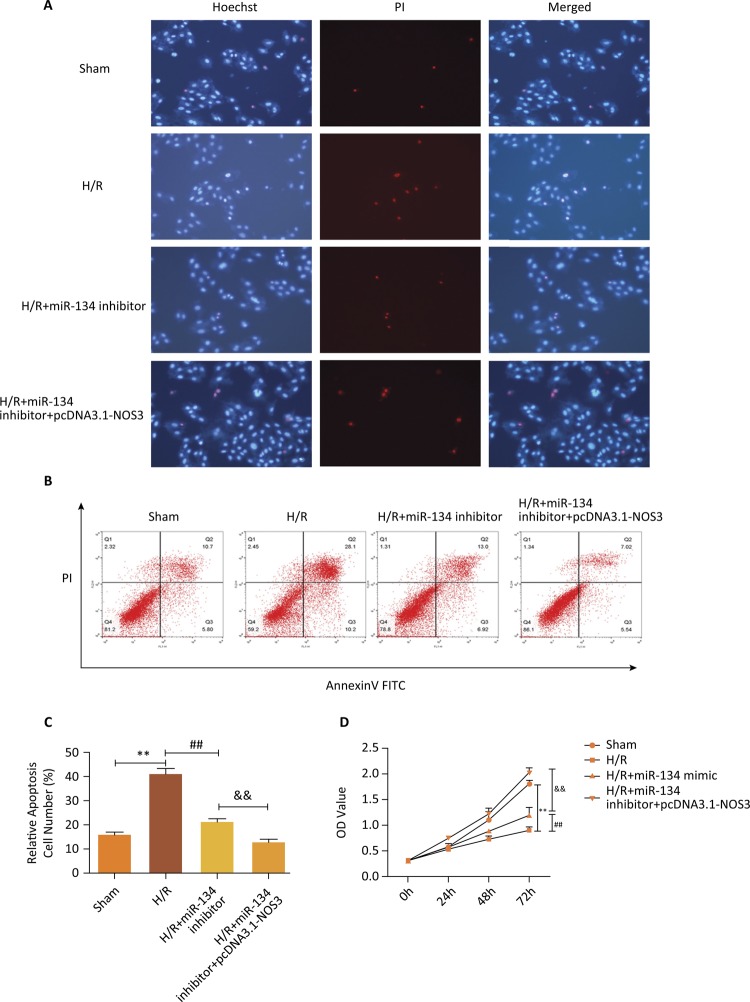
- NOS3 accentuated the relieve effect of miR-134 downregulation on myocardial damage resulted by H/R condition. ( A ) Apoptotic cell death was assessed using Hoechst 33342/PI double staining. ( B ) and ( C ) Cell apoptosis was detected by flow cytometry analysis, **P<0.01 *vs.* Sham group, ## P<0.01 *vs.* H/R group, && P<0.01 *vs.* H/R+miR-134 inhibitor group. ( D ) Detection of cell viability using CCK-8 assay, **P<0.01 *vs.* Sham group, ## P<0.01 *vs.* H/R group, && P<0.01 *vs.* H/R+miR-134 inhibitor group.

The next section was concerned with the influence of overexpressed NOS3 on the effect in cell apoptosis and growth induced by the miR-134 inhibitor. To confirm the apoptotic cell death, Hoechst 33342/PI double staining experiment was implemented. As shown in Figure 5A , H/R treatment contributed to the death of apoptotic cells compared with the Sham group, and miR-134 inhibitor and overexpression of NOS3 significantly inhibited the death induced by H/R treatment. Cell apoptosis assay elucidated that miR-134 downregulation led to visible suppression in apoptotic ability followed by H/R treatment; likewise, high expression of NOS3 strengthened the decreased number of cell apoptosis that resulted of the miR-134 inhibitor interference ( Fig. 5B-C , P<0.01). CCK-8 analysis implied that upregulated NOS3 provided the consistent function with miR-134 inhibitor on cell viability in H9c2 cells treated by H/R condition. All they can promote cell proliferative capability when the H/R group was considered as control ( Fig. 5D , P<0.01). Overall, these results validated that NOS3 is a target gene of miR-134 and its overexpression could stress the mitigation of miR-134 on myocardial damage.

## Discussion

It has been extensively studied that H/R can trigger damage to the membrane of cardiomyocytes, further leading to release of cellular enzymes. Thus, the detection of LDH activity contributes to confirm the degree of myocardial damage ^14 , 15^ . In addition, considering the important significance of skeletal myoblasts in heart diseases, we selected H9c2 cells with better stability and reproducibility instead of primary rat cardiomyocytes ^16 , 17^ . MiR-134 is a well-described microRNA in brain, and it has been widely used to modulate the development and physiology of brain ^18^ . According to previous researches, we observed that miR-134 is located in hippocampal neurons in rats and interacts with memory formation through controlling CREB protein expression level ^19 , 20^ . In the study of Rong *et al* . ^21^ , miR-134 was presumed to be a peripheral biomarker for diagnosis of bipolar disorder because of its altered level in blood. This seems to be prompting us that miR-134 was involved in brain-related diseases by manipulating physiological reactions correlated with blood. As evidence, it has been verified that a reduction of miR-134 can moderate ischemic injury ^10^ . Mice were also utilized to demonstrate the effect of miR-134 in the cerebral ischemic cortex and similar results were obtained ^22^ . Based on the extensive literature about the involvement of miRNAs in myocardial I/R injury treatment and the impact of miR-134 in ischemic disorders, we turned to explore the effects of miR-134 against myocardial H/R injury ^22 - 24^ . As expected, we found that miR-134 was increased after H/R by means of H9c2 cells and the conduction of flow cytometry assay and CCK-8 analysis showed us that miR-134 mimic/inhibitor can strengthen/alleviate cardiomyocyte injury led by H/R. LDH activity was also regulated by a diverse expression level of miR-134.

To complete this study, we next predicted the target gene of miR-134 and discovered that NOS3 is one of the target genes of miR-134. NOS3 is a major gene, encoding endothelial NOS (eNOS). A considerable amount of literature has been published to illustrate the essential effect of eNOS for the protection of a normal cardiovascular system ^25^ . Its primary role is attributed to the generation of NO; and then the generated NO is used to regulate vascular tone, platelet aggregation and cell proliferation ^26^ . On the basis of the above mentioned, Zhang *et al* . ^27^ indicated that the activity of eNOS was encouraged by alprostadil to protect myocardial I/R injury. Protective impacts induced by Shenfu injection on myocardial H/R injury was supported by activated eNOS ^28^ . Here, we confirmed the repressive expression of NOS3 was linked with upregulated miR-134. Increased NOS3 could further redouble the miR-134-trigerred positive role on myocardial damage, including the inhibition of cellular apoptosis and promoting cell growth. In general, these observations pointed that miR-134 is responsible for the myocardial I/R injury mediating NOS3 level.

It is known to all that the PI3K/Akt pathway is an important signaling pathway diametrically correlated with cellular behaviors and diverse molecular mechanisms ^29^ . Moreover, in accordance with the previous reports, the myocardial I/R injury moderation has a close interaction with the PI3K/Akt/eNOS signaling pathway ^30^ , which is consistent with our results that higher expression of NOS3 was beneficial to myocardial I/R injury. A large set of significant clusters has also raised the credibility of the correlation between the PI3K/Akt pathway and myocardial I/R injury through diverse target substances, such as total paeony glycoside, hesperidin, shikonin, 6-Gingerol and so forth ^31 - 34^ . Therefore, we implemented western blot to depict the expressional level of marker proteins, p-PI3K and p-Akt, and discovered that the miR-134 inhibitor ameliorated the suppression of PI3K/Akt phosphorylation caused by H/R. This finding gave an account that the protective effect of down-regulated miR-134 on myocardial H/R injury was achieved by regulating the PI3K/Akt pathway.

## Conclusions

The deletion of miR-134 could attenuate myocardial H/R injury modulating the PI3K/Akt pathway and targeting NOS3, thus weakening apoptotic activity and elevating proliferative ability. These results will prove useful in expanding our knowledge of how to treat myocardial I/R injury effectively.

## Footnotes

1 Research was performed at Department of Cardiovascular Medicine, Daqing Oilfield General Hospital.

## References

[1] Chen WW, Gao RL, Liu LS, Zhu ML, Wang W, Wang YJ, Wu ZS, Li HJ, Gu DF, Yang YJ, Zheng Z, Jiang LX, Hu SS (2017). China cardiovascular diseases report 2015: a
summary. J Geriatr Cardiol.

[2] White HD, Chew DP (2008). Acute myocardial infarction. Lancet.

[3] Bangalore S, Pursnani S, Kumar S, Bagos PG (2013). Percutaneous coronary intervention versus optimal medical therapy
for prevention of spontaneous myocardial infarction in subjects with stable
ischemic heart disease. Circulation.

[4] Aghaei M, Motallebnezhad M, Ghorghanlu S, Jabbari A, Enayati A, Rajaei M, Pourabouk M, Moradi A, Alizadeh AM, Khori V (2019). Targeting autophagy in cardiac ischemia/reperfusion injury: a
novel therapeutic strategy. J Cell Physiol.

[5] Chi HJ, Chen ML, Yang XC, Lin XM, Sun H, Zhao WS, Qi D, Dong JL, Cai J (2017). Progress in therapies for myocardial ischemia reperfusion
injury. Curr Drug Targets.

[6] Minhas G, Mathur D, Ragavendrasamy B, Sharma NK, Paanu V, Anand A (2017). Hypoxia in CNS Pathologies: emerging role of miRNA-based
neurotherapeutics and yoga based alternative therapies. Front Neurosci.

[7] Wu G, Tan J, Li J, Sun X, Du L, Tao S (2019). miRNA-145-5p induces apoptosis after ischemia-reperfusion by
targeting dual specificity phosphatase 6. J Cell Physiol.

[8] Zhao YB, Zhao J, Zhang LJ, Shan RG, Sun ZZ, Wang K, Chen JQ, Mu JX (2019). MicroRNA-370 protects against myocardial ischemia/reperfusion
injury in mice following sevoflurane anesthetic preconditioning through
PLIN5-dependent PPAR signaling pathway. Biomed Pharmacother.

[9] Zhu XA, Gao LF, Zhang ZG, Xiang DK (2019). Down-regulation of miR-320 exerts protective effects on
myocardial I-R injury via facilitating Nrf2 expression. Eur Rev Med Pharmacol Sci.

[10] Huang W, Liu X, Cao J, Meng F, Li M, Chen B, Zhang J (2015). miR-134 regulates ischemia/reperfusion injury-induced neuronal
cell death by regulating CREB signaling. J Mol Neurosci.

[11] Zhou J, Chen L, Chen B, Huang S, Zeng C, Wu H, Chen C, Long F (2018). Increased serum exosomal miR-134 expression in the acute ischemic
stroke patients. BMC Neurol.

[12] Wu YH, Zhao H, Zhou LP, Zhao CX, Wu YF, Zhen LX, Li J, Ge DX, Xu L, Lin L, Liu Y, Liang DD, Chen YH (2015). miR-134 Modulates the proliferation of human cardiomyocyte
progenitor cells by targeting meis2. Int J Mol Sci.

[13] Wang KJ, Zhao X, Liu YZ, Zeng QT, Mao XB, Li SN, Zhang M, Jiang C, Zhou Y, Qian C, Feng KG, Guan HQ, Tang TT, Cheng X, Chen ZJ (2016). Circulating MiR-19b-3p, MiR-134-5p and MiR-186-5p are promising
novel biomarkers for early diagnosis of acute myocardial
infarction. Cell Physiol Biochem.

[14] Li Y, Li Q, Zhang O, Guan X, Xue Y, Li S, Zhuang X, Zhou B, Miao G (2019). miR-202-5p protects rat against myocardial ischemia reperfusion
injury by downregulating the expression of Trpv2 to attenuate the Ca (2+)
overload in cardiomyocytes. J Cell Biochem.

[15] Du XJ, Wei J, Tian D, Yan C, Hu P, Wu X, Yang W, Hu X (2019). NEAT1 promotes myocardial ischemia-reperfusion injury via
activating the MAPK signaling pathway. J Cell Physiol.

[16] Hayashi E, Hosoda T (2014). Myocyte renewal and therapeutic myocardial regeneration using
various progenitor cells. Heart Fail Rev.

[17] Chen CH, Sereti KI, Wu BM, Ardehali R (2015). Translational aspects of cardiac cell therapy. J Cell Mol Med.

[18] Ma Q, Zhang L, Pearce WJ (2019). MicroRNAs in brain development and cerebrovascular
pathophysiology. Am J Physiol Cell Physiol.

[19] Tai HC, Schuman EM (2006). MicroRNA: microRNAs reach out into dendrites. Curr Biol..

[20] Gao J, Wang WY, Mao YW, Graff J, Guan JS, Pan L, Mak G, Kim D, Su SC, Tsai LH (2010). A novel pathway regulates memory and plasticity via SIRT1 and
miR-134. Nature.

[21] Rong H, Liu TB, Yang KJ, Yang HC, Wu DH, Liao CP, Hong F, Yang HZ, Wan F, Ye XY, Xu D, Zhang X, Chao CA, Shen QJ (2011). MicroRNA-134 plasma levels before and after treatment for bipolar
mania. J Psychiatr Res.

[22] Chi W, Meng F, Li Y, Wang Q, Wang G, Han S, Wang P, Li J (2014). Downregulation of miRNA-134 protects neural cells against
ischemic injury in N2A cells and mouse brain with ischemic stroke by
targeting HSPA12B. Neuroscience.

[23] Chi W, Meng F, Li Y, Li P, Wang G, Cheng H, Han S, Li J (2014). Impact of microRNA-134 on neural cell survival against ischemic
injury in primary cultured neuronal cells and mouse brain with ischemic
stroke by targeting HSPA12B. Brain Res.

[24] Liu W, Wu J, Huang J (2017). Electroacupuncture regulates hippocampal synaptic plasticity via
miR-134-mediated LIMK1 function in rats with ischemic Stroke.

[25] Joshaghani HR, Salehi A, Samadian E, Gharaei R, Ahmadi AR (2018). Association between NOS3 G894T, T-786C and 4a/4b Variants and
coronary artery diseases in iranian population. Iran J Public Health.

[26] Sumpio BE, Riley JT, Dardik A (2002). Cells in focus: endothelial cell. Int J Biochem Cell Biol.

[27] Zhang L, Zhang Y, Yu X, Xu H, Sui D, Zhao X (2018). Alprostadil attenuates myocardial ischemia/reperfusion injury by
promoting antioxidant activity and eNOS activation in rats. Acta Cir Bras.

[28] Wang YY, Li YY, Li L, Yang DL, Zhou K, Li YH (2018). Protective effects of shenfu injection against myocardial
ischemia-reperfusion injury via activation of eNOS in rats. Biol Pharm Bull.

[29] Chen L, Qin L, Liu X, Meng X (2019). CTRP3 alleviates Ox-LDL-induced inflammatory response and
endothelial dysfunction in mouse aortic endothelial cells by activating the
PI3K/Akt/eNOS pathway. Inflammation.

[30] Liu Q, Li Z, Liu Y (2018). Hydromorphine postconditioning protects isolated rat heart
against ischemia-reperfusion injury via activating P13K/Akt/eNOS
signaling. Cardiovasc Ther.

[31] Shen P, Chen J, Pan M (2018). The protective effects of total paeony glycoside on
ischemia/reperfusion injury in H9C2 cells via inhibition of the PI3K/Akt
signaling pathway. Mol Med Rep.

[32] Li X, Hu X, Wang J, Xu W, Yi C, Ma R, Jiang H (2018). Inhibition of autophagy via activation of PI3K/Akt/mTOR pathway
contributes to the protection of hesperidin against myocardial
ischemia/reperfusion injury. Int J Mol Med.

[33] Wang S, Zhu Y, Qiu R (2018). Shikonin protects H9C2 cardiomyocytes against
hypoxia/reoxygenation injury through activation of PI3K/Akt signaling
pathway. Biomed Pharmacother.

[34] Lv X, Xu T, Wu Q (2018). 6-Gingerol activates PI3K/Akt and inhibits apoptosis to attenuate
myocardial ischemia/reperfusion injury. Evid Based Complement Alternat Med.

